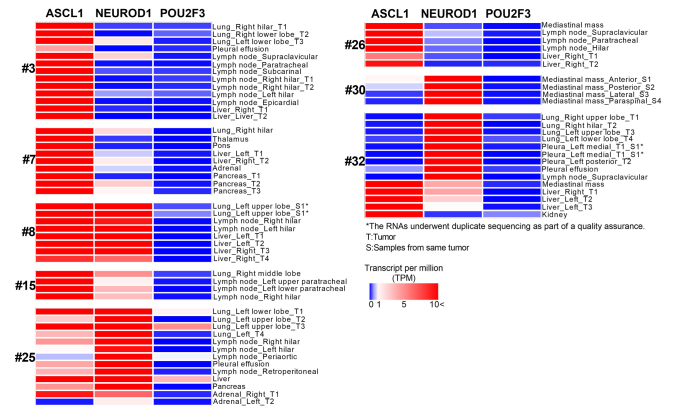# Unveiling the Molecular Features of SCLC With a Clinical RNA Expression Panel

**DOI:** 10.1016/j.jtocrr.2024.100723

**Published:** 2024-08-28

**Authors:** Hilal Ozakinci, Aileen Y. Alontaga, Pedro Cano, John M. Koomen, Bradford A. Perez, Amer A. Beg, Alberto A. Chiappori, Eric B. Haura, Theresa A. Boyle

**Affiliations:** a Department of Thoracic Oncology, H. Lee Moffitt Cancer Center & Research Institute, Tampa, Florida; b Department of Pathology, H. Lee Moffitt Cancer Center & Research Institute, Tampa, Florida; c Department of Molecular Oncology, H. Lee Moffitt Cancer Center & Research Institute, Tampa, Florida; d Department of Radiation Oncology, H. Lee Moffitt Cancer Center & Research Institute, Tampa, Florida; e Florida Cancer Specialists & Research Institute, Trinity Cancer Center, Trinity, Florida; f Department of Immunology, H. Lee Moffitt Cancer Center & Research Institute, Tampa, Florida

**Keywords:** Small cell lung cancer, RNA expression, Biomarker, Clinical testing, Transcriptomics

## Abstract

**Introduction:**

The translation of gene expression profiles of SCLC to clinical testing remains relatively unexplored. In this study, gene expression variations in SCLC were evaluated to identify potential biomarkers.

**Methods:**

RNA expression profiling was performed on 44 tumor samples from 35 patients diagnosed with SCLC using the clinically validated RNA Salah Targeted Expression Panel (RNA STEP). RNA sequencing (RNA-Seq) and immunohistochemistry were performed on two different SCLC cohorts, and correlation analyses were performed for the *ASCL1*, *NEUROD1*, *POU2F3*, and *YAP1* genes and their corresponding proteins. RNA STEP and RNA-Seq results were evaluated for gene expression profiles and heterogeneity between SCLC primary and metastatic sites. RNA STEP gene expression profiles of independent SCLC samples (n = 35) were compared with lung adenocarcinoma (n = 160) and squamous cell carcinoma results (n = 25).

**Results:**

The RNA STEP results were highly correlated with RNA-Seq and immunohistochemistry results. The dominant transcription regulator by RNA STEP was *ASCL1* in 74.2% of the samples, *NEUROD1* in 20%, and *POU2F3* in 2.9%. The *ASCL1*, *NEUROD1*, and *POU2F3* gene expression profiles were heterogeneous between primary and metastatic sites. SCLCs displayed markedly high expression for targetable genes *DLL3*, *EZH2*, *TERT*, and *RET*. SCLCs were found to have relatively colder immune profiles than lung adenocarcinomas and squamous cell carcinomas, characterized by lower expression of *HLA* genes, immune cell, and immune checkpoint genes, except the *LAG3* gene.

**Conclusions:**

Clinical-grade SCLC RNA expression profiling has value for SCLC subtyping, design of clinical trials, and identification of patients for trials and potential targeted therapy.

## Introduction

SCLC is a dismal malignancy compromising approximately 14% of all lung cancers (National Comprehensive Cancer Network v.2.24-November 21, 2023). Treating SCLC presents a formidable challenge for oncologists, and the outcomes of treatment remain unsatisfactory. Elucidation of predictive biomarkers, development of better methods for selecting immunotherapy-sensitive populations,^1^^,^^2^ and improvement in therapeutic strategies are needed. In select instances, molecular profiling is currently considered for patients with extensive-stage (ES) SCLC who fall into rare categories, such as those who have minimal tobacco exposure (never smoked to less than 10 cigarettes/d). In addition, molecular profiling may be considered in cases where there is a diagnostic challenge, because additional molecular information may influence the treatment approach (National Comprehensive Cancer Network v.2.24-November 21, 2023).

Developing a deeper understanding of SCLC biology through genomic characterization offers the potential for critically needed advancement in the care and treatment of patients with SCLC. SCLC is characterized by inactivating mutations in the *RB1* and *TP53* tumor-suppressor genes with these mutations also associated with increased risk for SCLC transformation when identified in *EGFR*-mutant lung cancers. The neuroendocrine differentiation associated with SCLC is regulated by INSM1 which in turn is regulated by the Notch1-Hes1 signaling pathway.^3^ Early studies focusing on proteogenomic characterization defined four SCLC molecular subtypes based on high protein expression of the following markers: ASCL1 (SCLC-A), NEUROD1 (SCLC-N), POU2F3 (SCLC-P), and YAP1 (SCLC-Y).^4^^,^^5^ Later studies confirmed SCLC-A, SCLC-N, and SCLC-P as three distinct subtypes but did not validate the SCLC-Y as a distinct subtype.^6^, ^7^, ^8^ These distinct subtypes correlate with therapeutic responsiveness, differences in genetic alterations, and prognosis.^4^^,^^9^^,^^10^ In addition, some studies have presented compelling evidence for the existence of an SCLC inflamed subtype (SCLC-I) with lower expression of the *ASCL1*, *NEUROD1*, and *YAP1* genes, characterized by an inflamed gene signature and mesenchymal features.^10^ Although these foundational studies have made strides in the genomic characterization of SCLC, there remains an unmet need for the identification of biomarkers and new therapy targets for SCLC.

In this study, the primary objective was to explore SCLC gene expression variations with a clinically validated 204 gene expression panel, the RNA Salah Targeted Expression Panel (RNA STEP), to identify subtypes and potential diagnostic, prognostic, and therapeutic biomarkers.

## Materials and Methods

### Samples

**Figure 1:**
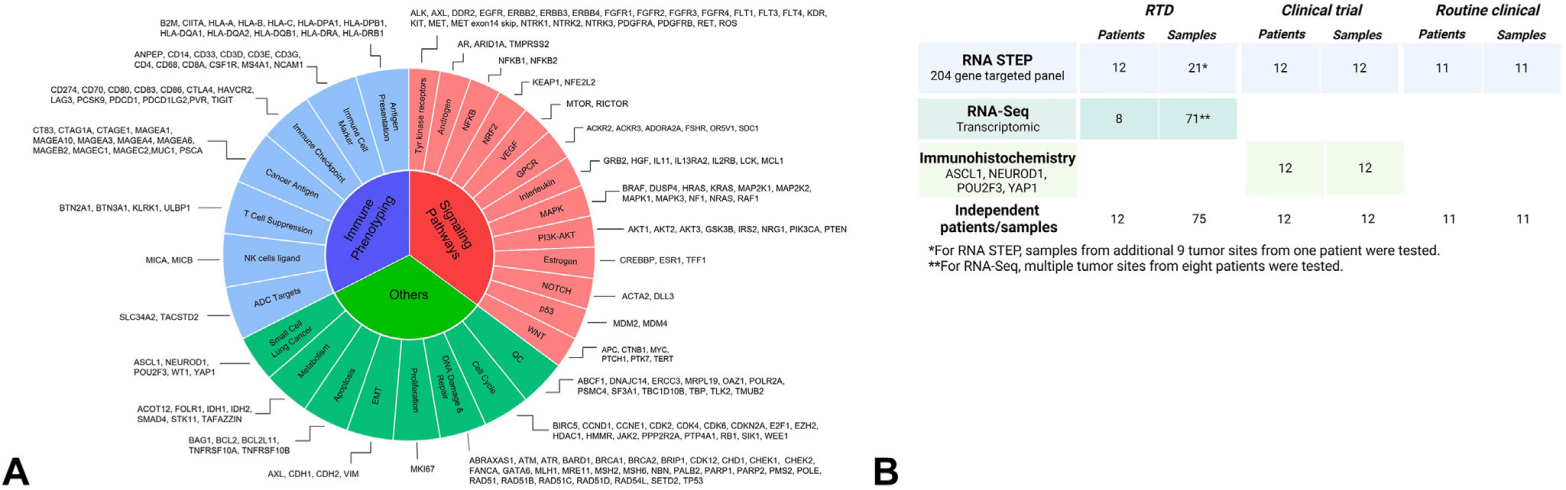
(*A*) RNA STEP. Panel targets include 204 genes. (*B*) Overview of the samples and methods included in the study. RNA STEP, RNA Salah Targeted Expression Panel.

Between November 2022 and November 2023, a total of 589 samples from solid tumors underwent RNA STEP testing (Fig. 1*A*), including samples from patients with a diagnosis of SCLC (n = 35), lung adenocarcinoma (LUAD, n = 160), and lung squamous cell carcinoma (SqCC, n = 25). This project to study deidentified RNA STEP results from routine clinical care was reviewed by the Advarra Institutional Review Board (IRB, IRB#00000971, MCC 23158) to help ensure that the rights and welfare of research participants were protected. The IRB granted a full waiver of the Health Insurance Portability and Accountability Act (HIPAA) authorization due to the impracticality of gaining retrospective consent from the many patients associated with the clinical results and the low ethical risk due to the deidentified nature of this study. Of the 35 SCLC samples, 11 were collected per routine clinical care. The remaining 24 were obtained through two IRB-approved research protocols: MCC19163 (n = 12), a vaccine-based clinical trial, and MCC18843 (n = 12), the rapid tissue donation (RTD) autopsy research protocol (Table 1, Fig. 1*B*). The vaccine-based clinical trial was registered on the ClinicalTrials.gov website (NCT00617409). All patients provided a written informed consent, and the treatment protocol was approved by University of South Florida Institutional Review Board. For the RTD study, in all cases where it was feasible, discussion of the study and informed consent was provided during clinical care.^11^ In addition, to evaluate for intermetastatic heterogeneity, nine additional samples from different tumor sites of one RTD donor (#32) were tested with RNA STEP.

**Table 1:** Demographics and Clinical Features of SCLC Cohort Tested by RNA STEP (n = 35)

Case Number	Age	Sex	Smoking History	Sample Type	Therapy-Naive Sample	Tested Tissue	Tumor Percentage
1	79	F	Former	Clinical	Yes	Liver	70
2	75	M	Former	Trial	No	Liver	15
3	57	F	Former	RTD	No	Lung	90
4	59	M	Current	Clinical	No	Liver	70
5	47	M	Former	Trial	No	Adrenal	90
6	67	F	Current	RTD	No	Lung	95
7	77	M	Former	RTD	No	Lung	90
8	69	M	Former	RTD	No	Lung	85
9	60	M	Former	Trial	No	Liver	80
10	59	M	Former	RTD	No	Liver	80
11	69	F	Former	Clinical	No	Liver	60
12	56	F	Former	Trial	No	Liver	90
13	66	F	Former	Trial	No	Liver	40
14	54	F	Former	RTD	No	Lung	90
15	74	M	Current	RTD	No	Lymph node	20
16	59	F	Former	Trial	No	Lung	60
17	74	F	Current	Trial	No	Lymph node	90
18	66	F	Former	Trial	No	Adrenal	70
19	63	M	Former	RTD	No	Liver	90
20	66	F	Current	Clinical	Yes	Lymph node	30
21	74	M	Current	Clinical	Yes	Retroperitoneum	60
22	77	M	Former	Trial	No	Liver	60
23	77	F	Former	Clinical	No	Liver	50
24	68	M	Former	Clinical	Yes	Pleural	95
25	88	M	Former	RTD	No	Lung	95
26	78	M	Former	RTD	No	Lung	10
27	70	M	Former	Clinical	No	Lung	70
28	36	M	Nonsmoker	Clinical	Yes	Lung	75
29	72	F	Nonsmoker	Clinical	No	Lung	60
30	68	M	Current	RTD	Yes	Mediastinum	30
31	59	F	Former	Trial	No	Liver	60
32	59	M	Current	RTD	No	Lung	90
33	69	F	Current	Clinical	Yes	Lymph node	30
34	73	F	Former	Trial	No	Liver	70
35	70	M	Former	Trial	No	Soft tissue	60

*Note:* All SCLC samples were tested with RNA STEP (N = 35); trial samples were also tested with IHC (N = 12); eight of above 12 RTD samples were also tested with RNA-seq.

F, female; IHC, immunohistochemistry; M, male; RNA-seq, RNA sequencing; RNA STEP, RNA Salah Targeted Expression Panel; RTD, rapid tissue donation.

RNA sequencing (RNA-Seq) was performed on tumor from 71 SCLC samples from eight RTD donors. RNA STEP was performed on 17 of these samples, one sample each from seven RTD donors and 10 samples from one donor (#32). There were 12 samples included in this study from a cohort of 21 patients with SCLC enrolled for a clinical trial with sufficient tissue for both immunohistochemical (IHC) staining and RNA STEP.

### RNA STEP

A custom RNA expression panel of 204 genes, RNA STEP, was designed with feedback elicited from Moffitt clinicians about which proteins or biomarkers would be most relevant for future clinical trials. Probes were designed to target the correlative genes to these selected proteins. The RNA STEP assay used the NanoString platform to digitally count the mRNA transcripts in the samples. Each run included a reference universal mRNA control derived from pooled human normal tissues (BioChain Institute, Inc., Newark, CA, Catalog number R4234565) to ensure batch-to-batch consistency and normalize gene signals. Raw data files were processed and normalized using geNorm in the NanoString nSolver 4.0 advanced analysis software. The normalized log_2_ ratios were computed by subtracting the normalized log_2_ counts of the universal mRNA control genes from the log_2_ counts of the individual samples. In the RNA STEP validation, a log_2_ ratio of more than or equal to 2 was chosen as the criterion to define “high” expression.^12^ Nevertheless, considering that one SCLC sample had NEUROD1 protein expression by IHC, had a log_2_ ratio of 1.6 in the RNA STEP analysis, a log_2_ ratio cutoff of more than or equal to 1 was selected for designating “high” expression in this study. For *ASCL1*, *NEUROD1*, *POU2F3*, and *YAP1*, the dominant transcription regulator was identified as the gene exhibiting the highest log_2_ ratio in the sample.

The methodologies for RNA extraction, RNA-Seq, IHC, and statistical analyses are provided in Supplementary Data 1.

## Results

### SCLC Cohort Demographics and Clinical Features

Of the 35 patients with SCLC included in this study, the mean age was 67 years, 54.2% (19/35) were male, 45.8% (16/35) were female, and 94.3% (33 of 35) had a history of current or former smoking with two patients having a nonsmoking history (Table 1). During clinical care, 94.3% (33/35) patients had undergone systemic therapy, including chemotherapy and immunotherapy, and the remaining 5.7% (2/35) had not received systemic treatment. Of 35 independent samples tested, 20% (7/35) were therapy-naive samples. The distribution of analyzed tissue sites was as follows: 37.1% (13/35) liver, 34.2% (12 of 35) lung, 11.4% (4/35) lymph nodes, 5.7% (2/35) adrenal glands, 2.9% (1/35) pleura, 2.9% (1/35) mediastinum, 2.9% (1/35) retroperitoneum, and 2.9% (1/35) soft tissue. The mean tumor percentage in the analyzed samples was 66.3% (range 10%–95%). In the clinical diagnostic samples, tumors from 94.3% (33/35) patients had positive expression for at least one neuroendocrine marker (synaptophysin, chromogranin A, CD56, INSM1) by IHC. In the remaining two patients (#12 and #34), the IHC results were unavailable for one (#12) and only CD56 IHC was performed in the other (#34) with positivity only in rare tumor cells.

### Comparison of RNA STEP Results With RNA-Seq and IHC

**Figure 2:**
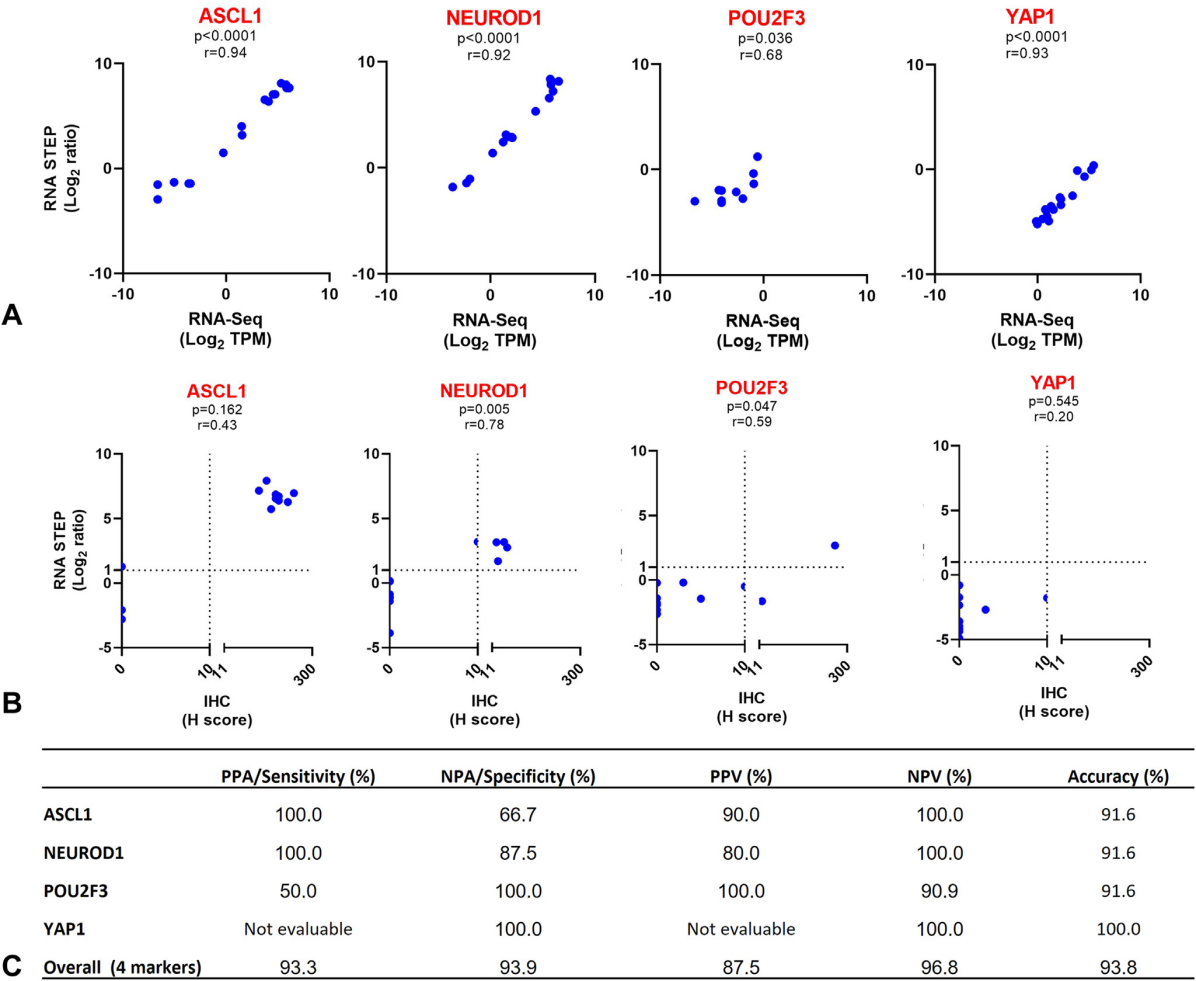
Comparison of RNA STEP, RNA-Seq, and IHC results. (*A*) RNA STEP and RNA-Seq revealed a statistically significant correlation for four transcriptional factors of SCLC (n = 17, *p*-values all <0.05). Numbers in the x and y axis are illustrated as log_2_ values of RNA-Seq TPM values and log_2_ ratio of RNA STEP, respectively. (*B*) Statistically significant correlations were observed in the comparison of RNA STEP (log_2_ ratio) and IHC (H score) results for the *NEUROD1* and *POU2F3* genes (n = 12, *p* < 0.05). (*C*) In the comparative analyses of RNA STEP and IHC results, the PPA or sensitivity reached 100% for the *ASCL1* and *NEUROD1* genes, whereas the NPA or specificity achieved 100% for the *POU2F3* and *NEUROD1* genes. The *YAP1* gene was not assessable for PPA or PPV due to the absence of any positive results. IHC, immunohistochemistry; NPA, negative percent agreement; PPA, positive percent agreement; RNA-Seq, RNA sequencing; RNA STEP, RNA Salah Targeted Expression Panel.

Correlation analyses were conducted to compare RNA STEP versus RNA-Seq results for *ASCL1*, *NEUROD1*, *POU2F3*, and *YAP1* gene expression using samples tested with both methods (Supplementary Data 2, 17 samples, eight patients). Comparisons were performed with values converted to a logarithmic scale of 2 (Fig. 2*A* and Supplementary Data 3A) and with the normalized RNA STEP linear counts versus RNA-Seq TPM (transcripts per million, Supplementary Data 3B). All comparisons for *ASCL1*, *NEUROD1*, and *YAP1* revealed statistically significant correlations with *p* values less than 0.0001 and r correlation coefficients more than 0.90. The *POU2F3* correlations were also statistically significant, though with weaker correlation values of r equal to 0.68 and *p* value equal to 0.036 for the normalized logarithmic comparison and r equal to 0.57 and *p* value equal to 0.018 for the normalized linear count raw data comparison.

To evaluate whether the RNA STEP results also correlated with protein expression, RNA STEP results were compared with protein expression by IHC for 12 samples (Supplementary Data 4). Despite the low number of samples, a significant correlation was observed between the RNA and protein expression for *NEUROD1* (N = 12, r = 0.78, *p* = 0.005) and *POU2F3* genes (r = 0.59, *p* = 0.047), but no significant correlation was observed for the *ASCL1* and *YAP1* genes (Fig. 2*B*). Concordance between the RNA STEP and IHC results was also evaluated using a log_2_ ratio cutoff of more than or equal to 1 and a H score cutoff of more than 10 to classify as positive for high gene and protein expression, respectively. The positive percent agreements (PPAs or sensitivity) for the *ASCL1*, *NEUROD1*, and *POU2F3* genes were 100%, 100%, and 50%, respectively. The *YAP1* gene could not be evaluated for PPA because there were no positive results by either assay. The negative percent agreements (NPAs or specificity) were 66.7%, 87.5%, 100%, and 100% for the *ASCL1*, *NEUROD1*, *POU2F3*, and *YAP1* genes, respectively. Overall, PPA and NPA for the four markers were 93.3% and 93.9%, respectively (Fig. 2*C*).

### Gene Expression Levels

**Figure 3:**
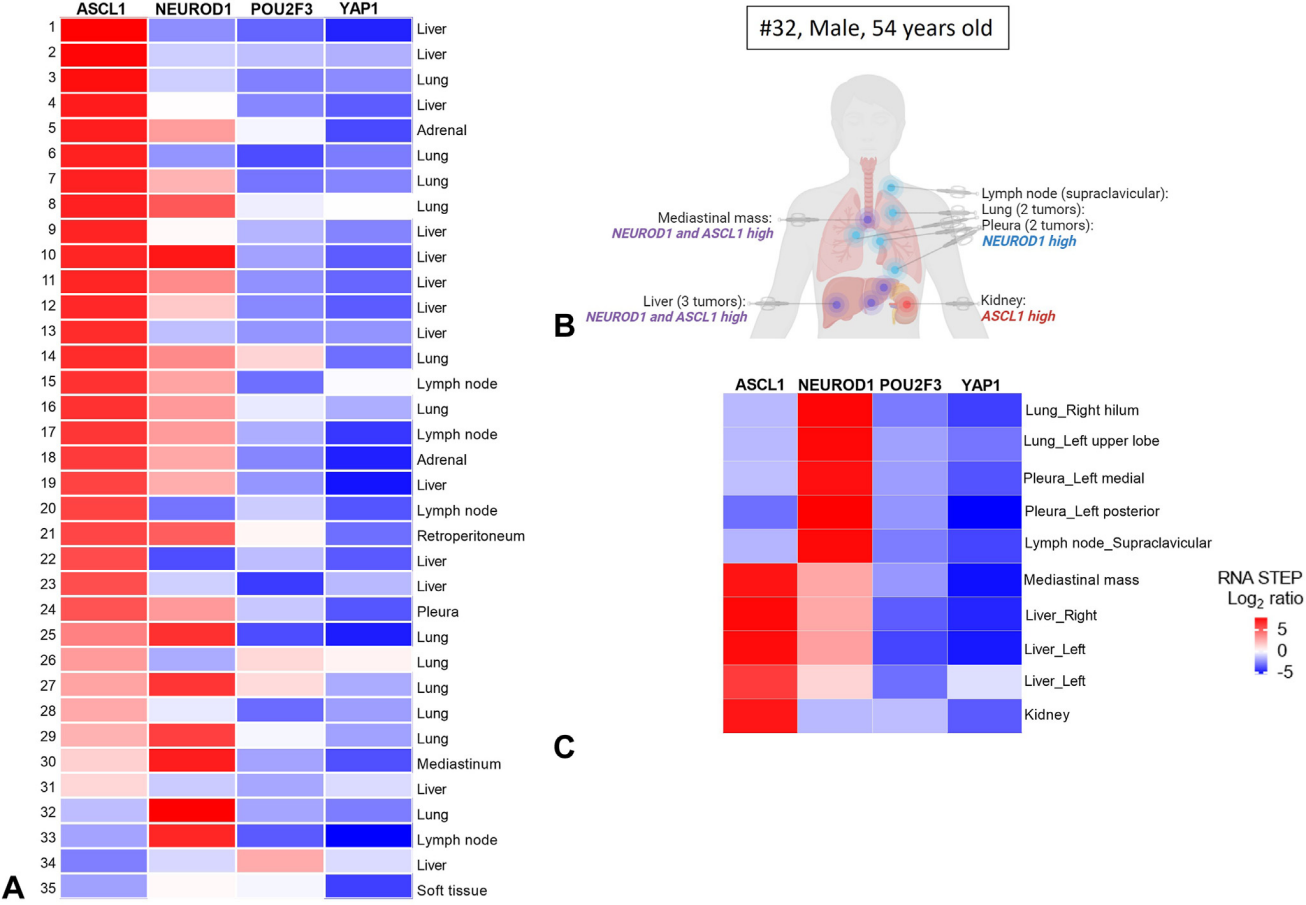
*ASCL1*, *NEUROD1*, *POU2F3*, and *YAP1* gene expression profile of SCLC. (*A*) RNA expression levels of *ASCL1*, *NEUROD1*, *POU2F3*, and *YAP1* genes in the tested 35 tumor samples by RNA STEP. RNA STEP results of samples 1 to 31 and 32 to 33 were ordered by highest to lowest *ASCL1* and *NEUROD1* results, respectively. (*B*, *C*) Comparison of *ASCL1*, *NEUROD1*, *POU2F3*, and *YAP1* gene expression levels in primary and metastatic tumors of same patient (#32, 54 y old, male) by RNA STEP. The distinct expression profiles observed in primary and metastatic tissues highlight substantial intrapatient heterogeneity. (*B*) Visual representation illustrating the gene expression profile of each tumor site. Created using BioRender.com. (*C*) Gene expression levels assessed by RNA STEP. RNA STEP, RNA Salah Targeted Expression Panel.

High expression of *ASCL1*, *NEUROD1*, and *POU2F3* genes was observed in 88.6% (31/35), 57.2% (20/35), and 11.4% (4/35) of the samples with RNA STEP, respectively. None of the samples had high *YAP1* gene expression (Fig. 3*A* and Supplementary Data 5).

In 42.8% (15/35) of the samples, high expression of only one of the transcription regulator genes was observed. In 60% (20/35) of the samples, high expression of two or three genes was observed as follows: 45.7% (16/35) samples with *ASCL1* and *NEUROD1* high gene expression, 2.9% (1/35) samples with *ASCL1* and *POU2F3* high gene expression, and 5.7% (2/35) samples with *ASCL1*, *NEUROD1*, and *POU2F3* high gene expression. One sample did not have high expression of any of these transcription regulation genes (Fig. 3*A* and Supplementary Data 5).

The transcription regulator gene with the most dominant (highest) expression was *ASCL1* in 74.2% (26/35), *NEUROD1* in 20% (7/35), and *POU2F3* in 2.9% (1/35) (Supplementary Data 6). Of note, in the one sample with dominant *POU2F3* gene expression (#34), only rare tumor cells had expression of CD56, a neuroendocrine marker, with IHC.

RNA STEP testing was performed successfully on 10 autopsy tumor samples from multiple tumor sites in one male RTD donor (#32) with SCLC. Tumors from the lungs, pleural tumors, and a supraclavicular lymph node had sole high *NEUROD1* expression. Both high *NEUROD1* and *ASCL1* expression was detected in the liver tumors and a mediastinal mass. By contrast, a more geographically distant kidney tumor had sole high *ASCL1* expression (Fig. 3*B* and *C*). Analyses of RNA-Seq results (Supplementary Data 7) on this patient revealed a similar pattern of positivity as the RNA STEP results.

RNA-Seq analyses of multiple tumor sites from eight RTD donors with SCLC highlighted the high prevalence of intermetastatic heterogeneity^13^ (#3, 7, 25, 32) for transcriptional regulator gene expression (Supplementary Data 7 and 8).

### SCLC, LUAD, and SqCC Gene Expression Profiles

**Figure 4:**
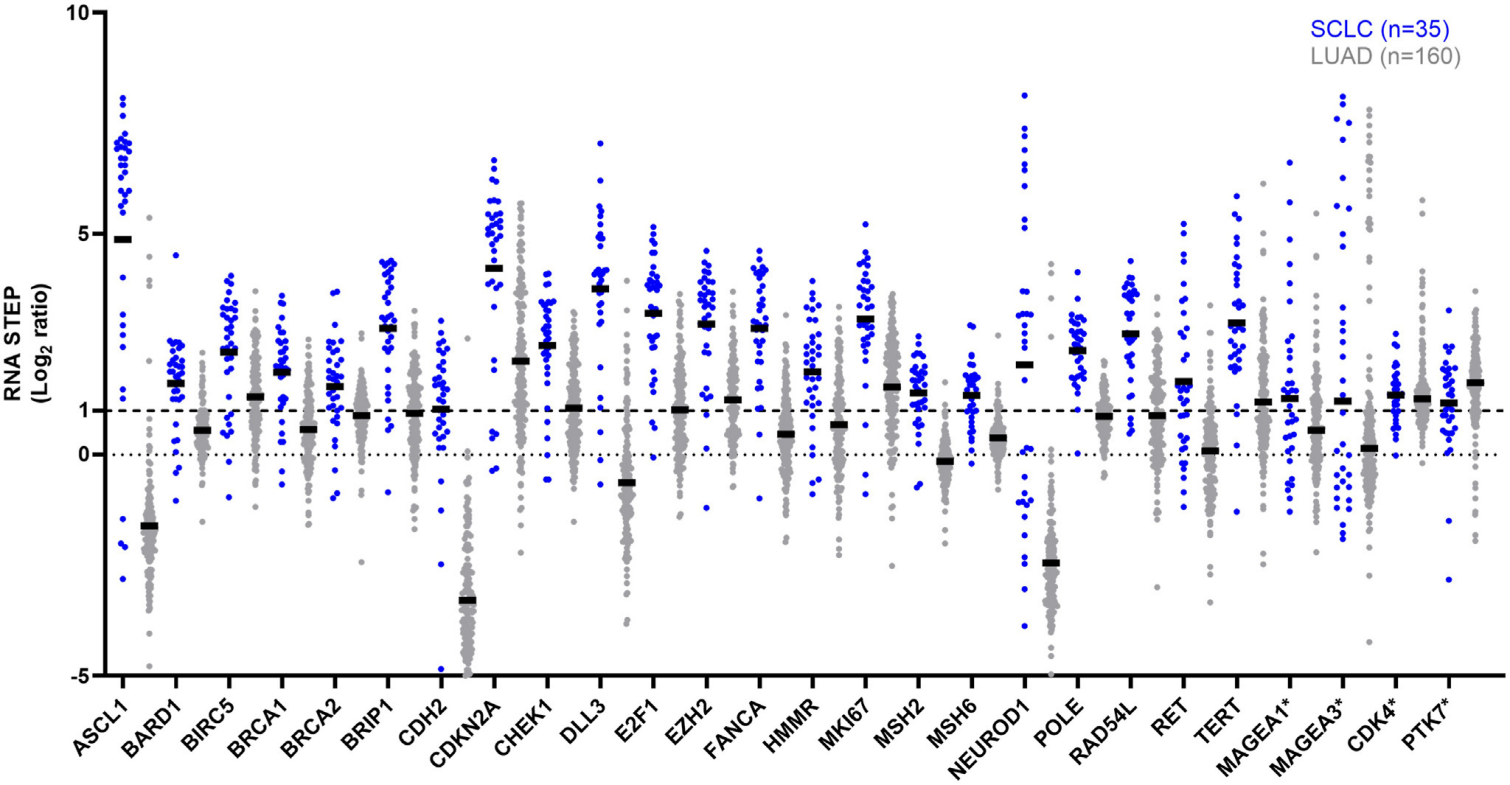
Gene expression analysis in SCLC and LUAD. RNA STEP revealed high expression of 26 genes in SCLC (mean log_2_ ratio ≥ 1 by RNA STEP). Among these, 22 genes exhibited significantly higher expression in SCLC compared with LUAD (*p* < 0.0001). The black bars represent the mean values. ∗No statistically significant difference was detected for the *MAGEA1*, *MAGEA3*, *CDK4*, and *PTK74* genes between SCLC and LUAD. LUAD, lung adenocarcinoma; RNA STEP, RNA Salah Targeted Expression Panel.

Among the 204 genes analyzed for expression with RNA STEP, 26 genes exhibited a high mean gene expression in the 35 tested SCLC samples (mean log_2_ ratio ≥ 1) (Fig. 4, Supplementary Data 9). Many of these genes play crucial roles in diverse pathways, including the basic helix-loop-helix family of transcription factors, DNA damage and repair processes, cell cycle regulation, epithelial-mesenchymal transition, NOTCH signaling, WNT signaling, tyrosine kinases, cancer antigens, and a proliferation marker. Within this subset of 26 genes with high general expression in SCLC, 22 genes also had significantly (*p* < 0.0001) higher relative expression in SCLC compared with LUAD (Fig. 4).

**Figure 5:**
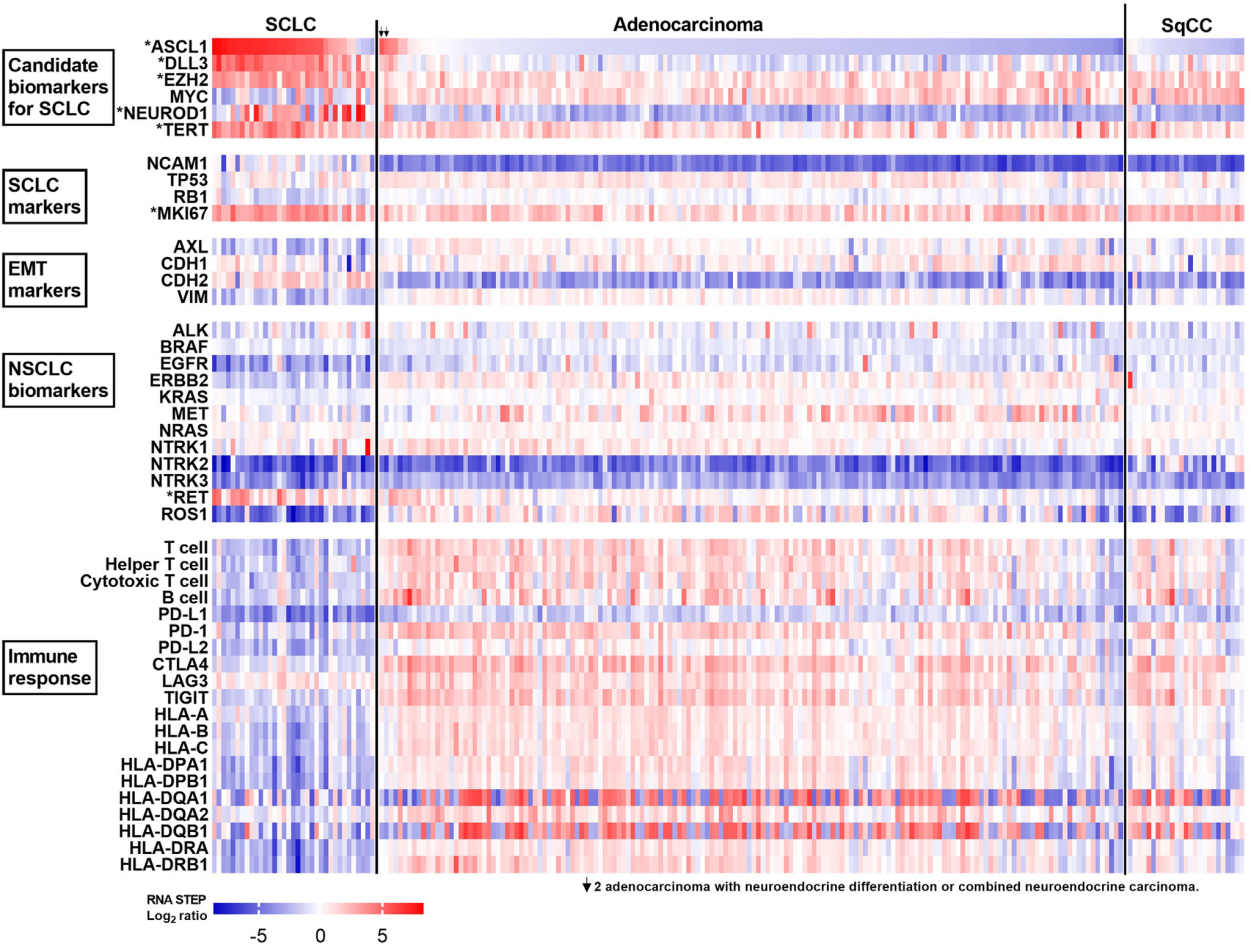
Gene expression profile of SCLC (n = 35), adenocarcinoma (n = 160), and SqCC (n = 25). ∗Genes exhibiting elevated expression level in SCLC (mean log_2_ ratio ≥) and having a statistically significant higher in expression in SCLC compared with LUAD (*p* < 0.0001). LUAD, lung adenocarcinoma; SqCC, squamous cell carcinoma.

Within the lung cancer cohort of 220 samples, high *ASCL1* expression was observed in 88.6% (31/35), 3.7% (6/160), and 0% (0/25) of the SCLC, LUAD, and SqCC samples, respectively. The clinicopathologic features of the six patients with LUAD with high *ASCL1* expression were reviewed. Neuroendocrine differentiation or combined neuroendocrine carcinoma was observed in two of the six samples (Fig. 5, arrows). Neuroendocrine differentiation was observed in the same biopsy sample of one patient, and the subsequent resection specimen from the other patient revealed a large cell neuroendocrine carcinoma component. High *NEUROD1* expression was found in 57.2% (20/35), 2.5% (4/160), and 4% (1/25) of the SCLC, LUAD, and SqCC samples, respectively. High *DLL3* and *EZH2* expression was observed in 91.4% (32/35) and 91.4% (32/35) in SCLCs, 11.2% (18/160) and 52.5% (84/160) in LUADs, and 24% (6/25) and 76% (19/25) in SqCCs, respectively (Fig. 5). SCLCs had high *TERT* expression in 91.4% (32/35) of samples versus 51.2% (82/160) in LUAD and 56% (14/25) in SqCC samples. High *CDKN2A* expression was observed in 85.8% (30/35) of the SCLC samples, and high *MYC* expression was found in 25.8% (9/35) of the SCLC samples (Fig. 5).

Tyrosine kinase genes associated with targeted therapy in NSCLC, such as *EGFR*, *ERBB2*, *MET*, and *ROS1*, had lower expression in SCLC than LUAD (Supplementary Data 9). In contrast, high *RET* expression was observed in 60% (21/35) of SCLC versus 16.9% (27/160) of LUAD and 4% (1/25) of SqCC samples. In addition, 93 genes had a mean log_2_ ratio less than 0 in SCLC and exhibited a significant decrease (*p* < 0.0001) in SCLC compared with LUAD (Supplementary Data 10). The SCLC cohort had generally lower expression of genes associated with T cells, helper T cells, cytotoxic T cells, B cells, and the *HLA* genes than the LUAD cohort, reflecting a generally “colder” immune microenvironment. Immune checkpoint markers, except *LAG3*, were also lower in the SCLC versus LUAD samples, suggesting a lower prevalence of immune cells and lower HLA antigen presentation in SCLC. Of 14 SCLC cases with high *LAG3* expression, none had concurrently high programmed death-ligand 1 (PD-L1 protein, *CD274* gene) expression, three had high programmed cell death protein-1 (PD-1 protein, *PDCD1* gene) expression, and three had high *CTLA4* expression, including one SCLC case with high *LAG3*, *PDCD1*, and *CTLA4* expression (Supplementary Data 11). The mean *LAG3* gene expression was similar between SCLC (mean log_2_ ratio = 0.72) and LUAD (mean log_2_ ratio = 0.97), despite the lower expression of immune cell markers in SCLC. The prevalence of samples with high *LAG3* expression was also similar between SCLC (40.0%, 14/35) and LUAD (51.2%, 82/160). In SCLC, LUAD, and SqCC, *LAG3* had a positive significant correlation with *PDCD1*, *CTLA4*, and *TIGIT,* despite gene expression values for *PDCD1*, *CTLA4*, and *TIGIT* being generally low in SCLC (all *p* < 0.05, Supplementary Data 12).

## Discussion

Therapeutic advancements for SCLC have historically fallen behind those for NSCLC. Nonetheless, in recent years, substantial strides have been made in understanding the molecular aspects of SCLC biology. Comprehensive genomic and transcriptomic analyses by tissue-based and blood-based testing have revealed molecular subtypes within SCLC categorized based on the gene expression levels of the following four transcriptional regulators: *ASCL1*, *NEUROD1*, *POU2F3*, and *YAP1*. Blood, unlike SCLC tissue, is easily available, and blood-based methods can rapidly provide molecular information from circulating biomarkers and cell free DNA/cell free RNA, making them ideal for ongoing monitoring.^14^^,^^15^ Nevertheless, blood-based testing may have limitations in sensitivity and specificity, especially when detecting low-abundance targets or in early stage disease. Future studies should aim to validate and compare blood- and tissue-based methods in lung cancer to fully establish their comparative effectiveness. In this study, the primary objective was to analyze gene expression variations, including *ASCL1*, *NEUROD1*, *POU2F3*, and *YAP1*, in SCLC with a clinical tissue-based targeted RNA expression panel, RNA STEP. Another aim was to gain a better understanding of the gene expression landscape of SCLC, especially genes associated with potential as diagnostic, prognostic, and therapeutic biomarkers.

RNA STEP was previously validated for clinical use with testing of more than 100 clinical samples. This study also aimed to evaluate the assay’s correlation for SCLC transcription regulator genes as compared with RNA-Seq and IHC results from the same samples. RNA STEP exhibited statistically significant correlations with RNA-Seq for *ASCL1*, *NEUROD1*, and *YAP1* (all p < 0.0001), and, though weaker, also for *POU2F3* (*p* = 0.036). RNA STEP and IHC results revealed 93.8% overall accuracy for all four markers. These findings underscore the ability of RNA STEP to provide accurate expression levels for these transcriptional regulators with the caveat that the sample size for these comparisons was small.

After assuring that the RNA STEP assay results were similar to RNA-Seq and IHC results, the prevalence of high *ASCL1*, *NEUROD1*, *POU2F3*, and *YAP1* gene expression in 35 SCLC samples with RNA STEP data was explored. High expression of the *ASCL1*, *NEUROD1*, and *POU2F3* genes was detected in 88.6%, 57.2%, and 11.4% of the SCLC samples, respectively. Consistent with previous studies,^16^ none of the SCLC samples had high *YAP1* expression in our cohort. The dominant transcription regulator was *ASCL1* in 74.2%, *NEUROD1* in 20%, and *POU2F3* in 2.9% of the samples (Supplementary Data 6). The average *ASCL1* gene expression was approximately fivefold higher in SCLC samples relatively to the pooled control sample (Supplementary Data 9). This observation aligns with the established role of the ASCL1 transcription factor as the master regulator in SCLC. The one sample with dominant *POU2F3* gene expression had neuroendocrine marker expression in only a few tumor cells by IHC consistent with a previous report describing the lower neuroendocrine character of tumors with high *POU2F3* levels.^17^ Of note, 54.2% of the samples had high expression of more than one transcription regulator gene. In the initial studies that delineated molecular subtypes, the occurrence of double/triple expression of transcription regulators was not reported. This may be attributed to the utilization of experimental models (human cell line models, genetically engineered animal models, and patient-derived xenografts) in these studies rather than human tumor samples which have a heterogeneous nature.^18^ In experimental models, there could be a clonal selection bias favoring one transcription regulator and suppressing others in the emerging monoclonal population, such that expression of only one marker is detected as opposed to the double/triple marker expression that may have been present in the native human samples.^19^

Furthermore, 50% of SCLCs may undergo subtype switching or exhibit a loss of expression of the ASCL1 and NEUROD1 transcription factors as the disease progresses.^20^^,^^21^ Rapid autopsies provide primary and metastatic samples to enable studies of both intratumoral and intermetastatic heterogeneity, including molecular changes during disease progression.^22^ In our study, a comparative analysis of RNA STEP results by testing primary and metastatic tumors from 12 RTD donors was conducted. Notably, for donor #32, lung and pleural tumors, along with a lymph node, displayed high gene expression of *NEUROD1* and low expression of *ASCL1*. Conversely, the mediastinal mass, liver, and kidney samples exhibited higher *ASCL1* than *NEUROD1* gene expression (Fig. 3*B* and *C*). In a previous study, loss of *ASCL1* expression was documented during progression^20^; conversely, our study reveals the loss of *NEUROD1* expression in distant metastatic sites.

RNA-Seq results provided additional support for the presence of transcription regulator intermetastatic heterogeneity (Supplementary Data 8). The heterogeneity of transcription regulator gene expression by tumor site suggests that a uniform treatment strategy based on transcription regulator subtype evaluation of one tumor site may not be optimal. For example, if a trial was designed to exclude patients with low ASCL1 expression and the lung from the patient associated with #32 was tested, this patient would be excluded from the trial, despite having high ASCL1 expression in the untested metastatic sites. Rather than designing trials based on transcription regulator subtypes, a more effective strategy might be to consider the most common general characteristics of SCLC and design trials with minimal exclusion criteria. The trials can aim to identify which patients might need a different therapeutic strategy. These results also provide evidence for the concept that different metastases may require different types of treatment, which is consistent with the common observation that some lesions regress or remain stable during therapy, but others progress.

RNA STEP results also provided a glimpse into the molecular landscape of SCLC. Of 204 genes covered by RNA STEP, 26 genes had high expression in more than half of the SCLC cases with 22 significantly higher expression in SCLC than LUAD (*p* < 0.0001). These genes included the major transcriptional regulators, *ASCL1* and *NEUROD1*, and *DLL3*, which is regulated by *ASCL1* (Fig. 4). The DLL3 protein is highly expressed on the cell surface of SCLC and plays a pivotal role as a negative regulator of NOTCH signaling.^19^^,^^23^ As of September 8, 2023, there are 327 clinical trials including patients with SCLC on the clinicaltrials.gov website.^1^ Of these, 22 specifically focus on *DLL3*, and within this subset, six are actively recruiting. The DeLLphi-301 trial in patients with ES SCLC reported an overall response rate of 40% to DLL3-targeted therapy tarlatamab-dlle (Imdelltra, Amgen, Inc.) leading to accelerated approval by the United States Food and Drug Administration (FDA) on May 16, 2024, which was biomarker agnostic.^24^ With RNA STEP, high *DLL3* expression was detected in 91.4% of SCLC samples, irrespective of *ASCL1* expression. Although some studies indicate higher *DLL3* expression in mainly the *ASCL1*-high group, others, including our study, also had high *DLL3* expression in *NEUROD1*-expressing SCLCs.^19^ The high prevalence of high *DLL3* gene expression in SCLC by RNA STEP corresponds with the high response rate to DLL3-targeted therapy in this patient population.

As a member of the PRC2 family, EZH2 serves as a transcription factor and activates trimethylation of H3K27me3, which alters the expression of downstream target genes resulting in cell proliferation, apoptosis, and senescence.^25^ Activation of the *EZH2* gene is associated with metastasis and reduced therapy response in lung cancer.^26^^,^^27^ Thus, EZH2 might be a therapeutic target to consider for patients with SCLC. A subset of 25.8% of the SCLC samples in this study had high *MYC* expression. The *MYC* family genes—*MYC*, *MYCL*, and *MYCN*—are recognized as oncogenic drivers and are considered potential biomarkers for SCLC.^28^ A trial targeting *MYC* could benefit from considering high *MYC* expression as an inclusion criteria; however, additional arms or sites might be needed to assure accrual if a similar cutoff for high expression is used. Comparative analysis of gene expression levels for key genes in SCLC versus LUAD revealed a similar prevalence of high *MYC* expression in SCLC and LUAD.

Comparative analyses of SCLC with LUAD and SqCC revealed a higher frequency of high *RET* expression in SCLC (60%) than LUAD (16.9%) or SqCC (4%), consistent with the findings of a study^29^ that reported 80% of SCLC cases had strong RET positivity by IHC. The *RET* gene encodes a transmembrane receptor, and the activation of this receptor triggers multiple oncogenic pathways. In 2022, FDA approved RET inhibitors for patients with *RET* fusion-positive solid cancers.^30^ In this study, 10 SCLC samples received previous clinical testing for *RET* fusions with clinical next-generation sequencing (Moffitt STAR, Illumina TSO500 platform). Although none of these samples had a *RET* fusion, six of 10 had elevated *RET* expression level by RNA STEP. One study reported that chromatin structure and promoter hypomethylation affect *RET* expression in cancer cells.^31^^,^^32^ As such, the high *RET* expression in our SCLC cohort might be associated with either copy number gains or epigenetic mechanisms. *RET* amplifications have been reported in many tumor types, including lung cancer.^33^ Platt et al^34^ reported that incidence of *RET* amplification is higher than rearrangements in NSCLC (2.8% versus 0.7%). Nevertheless, *RET* gene alterations have not been well studied in SCLC, and the clinical significance of *RET* copy number changes and their correlation with increased RET protein expression have not been well characterized yet.^33^

Other therapeutic targets for NSCLC, such as the *ALK*, *BRAF*, and *EGFR* genes, had lower expression in SCLC. In our cohort, high *TERT* expression was detected in 91.4% of SCLCs suggesting its potential as a therapeutic candidate for the treatment of SCLC. The *TERT* gene plays a key role in carcinogenesis by coding a protein which can prevent progressive shortening of telomeres by the reverse transcriptase activity.^35^ The results of this study are consistent with a previous study that reported elevated *TERT* expression level in SCLC, particularly in SCLCs that have undergone transformation from LUAD.^36^ To the best of our knowledge, the number of studies describing the high prevalence of *TERT* expression is limited. One study describes how abnormal methylation of *TERT* promotor leads to higher *TERT* expression which enhances the progression and radiotherapy resistance in SCLC.^37^ Another study suggests that TERT inhibitors (NU-1) can promote antitumor immunity after radiation.^38^

The survival of cancer cells and the effectiveness of therapy are heavily affected by the immune microenvironment. Nevertheless, our understanding of the immune microenvironment in SCLC and reliable biomarkers for predicting response to immunotherapy in SCLC remain elusive.^39^ Previous studies have found an association between survival postimmunotherapy with the infiltration of cytotoxic cells, coupled with high MHC-I expression in pretreatment samples from patients with SCLC.^40^ In accordance with other studies,^41^^,^^42^ our study revealed an immune cold profile in SCLC with low expression of markers for T lymphocytes, including CD4+ helper T cells and CD8+ cytotoxic T cells, immune checkpoint proteins (except the *LAG3* gene), and *HLA* genes. Although expression of genes associated with PD-1 and CTLA4 was generally lower than *LAG3* gene expression, their expression still correlated with *LAG3* expression. It may be that the lower overall prevalence of immune cells in SCLC causes a parallel lower level of immune checkpoint expression, though with continued expression of LAG3, PD-1, and CTLA4 on specific immune cells. We recognize that this RNA expression study provides only a glimpse of the immune cold environment of SCLC. Future studies, such as with special technologies like multiplex immunofluorescence, are needed to better elucidate the mechanisms that cause the low immunogenicity in SCLC. Mechanistic possibilities for the immune cold environment include impaired crosstalk between T cells and conventional-type dendritic cells and low expression of *HLA* genes.

The LAG3 protein, encoded by the *LAG3* gene, inhibits T cell responses by binding to stable peptide-MHC class II and is a promising target for immunotherapy.^43^^,^^44^ In our cohort, 40% of SCLCs (14 of 35) had high *LAG3* expression. This finding aligns with analysis of a public data set,^45^ which described elevated *LAG3* expression level in SCLC compared with normal lung tissue. These results underscore the potential of LAG3 as a candidate biomarker in SCLC, supporting the importance of clinical trials to study the potential for LAG3 checkpoint inhibition (NCT03219268, NCT03365791, NCT03538028). Of note, inhibition of LAG3 may be necessary, but insufficient alone, to ignite immune activation against the tumor, especially with the low expression of HLA genes in SCLC. A therapeutic strategy that does not require HLA antigen presentation may be needed for immune activation. Anti-LAG3 plus a bispecific T cell engager targeting an SCLC surface protein might activate T cells without relying on HLA antigen presentation.^46^

### Conclusion

The complementary addition of clinical RNA expression profiling in SCLC to established diagnostic techniques provides support for molecular diagnostic classification. The implementation of RNA expression profiling of SCLC clinical samples harnesses the opportunity to inform clinical trial design and translational biomarker studies. The RNA STEP has an extraction-free, simple, and rapid workflow of 3 days which works well with FFPE samples that often have degraded and low RNA yield and offers potential for a comprehensive perspective on the molecular distinctions within tumors. This panel may unveil novel biomarkers, identify prospects for clinical trials, and present therapeutic opportunities for the management of SCLC.

## CRediT Authorship Contribution Statement

**Hilal Ozakinci:** Conceptualization, investigation, sample collection, methodology, project supervision, pathology review, data analysis, writing-original draft, review and editing.

**Aileen Y. Alontaga:** Methodology-RNA STEP, writing-review and editing.

**Pedro Cano:** Developed the informatics pipeline of RNA STEP.

**John M. Koomen:** Supervision, Writing-Review and editing.

**Bradford A. Perez:** Methodology-RNA-Seq, Supervision, Data Acquisition, Writing-review and editing.

**Amer A. Beg:** Data analysis, supervision, writing-review and editing.

**Alberto A. Chiappori:** Funding Acquisition, Conceptualization, Writing-Review and editing.

**Eric B. Haura:** Project conceptualization, Supervision, Funding Acquisition, Writing-Review and editing.

**Theresa A. Boyle:** Project conceptualization, funding acquisition, methodology-RNA STEP, data analysis, supervision, writing-original draft, review and editing.

All authors read and approved the final manuscript.

## Study Approval

This work has been carried out in accordance with The Code of Ethics of the World Medical Association (Declaration of Helsinki) for experiments involving humans. Tissue samples were procured in line with WHO Guiding Principles on Human Cell, Tissue and Organ Transplantation. Clinical trial and rapid tissue donation were approved by the Institutional Review Board (Advarra, Columbia, MD, Pro00014653 and Pro00030829, respectively). RNA STEP analyses were conducted with a study protocol exemption (MCCC23158, Pro00076403).

## Research Data/Data Availability

The data that support the findings of this study are available from the corresponding authors on reasonable request.

## Submission Declaration and Verification

This work is not under consideration for publication elsewhere.

## Disclosure

Dr. Chiappori received funding from 10.13039/100002491Bristol-Myers Squibb for the clinical trial (MCC19163). Dr. Boyle and Dr. Koomen declare grants/contracts with Bristol-Myers Squibb unrelated to this research. Dr. Perez declares receiving grants from Bristol-Myers Squibb; providing consulting services for AstraZeneca, Bristol-Myers Squibb, G1 Therapeutics, and Novocure; and having board membership in Out of Zion. Dr. Haura declares providing consulting services for Kanaph Therapeutics and ORI Capital II; receiving research funding from 10.13039/100019364Revolution Medicines; and providing advisory services for RevMed and Janssen, all unrelated to this research. The remaining authors declare no conflict of interest.

## Figures and Tables

**Supplementary Data 10:**
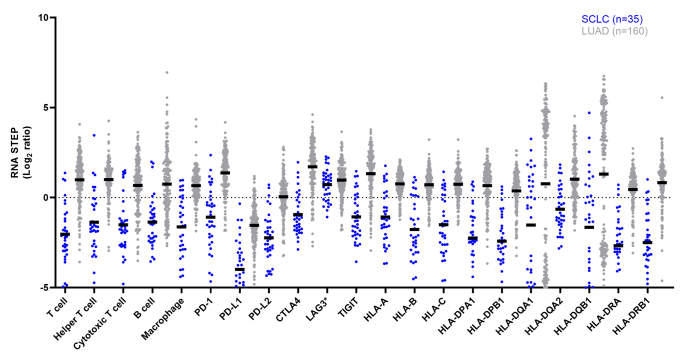


**Supplementary Data 12:**
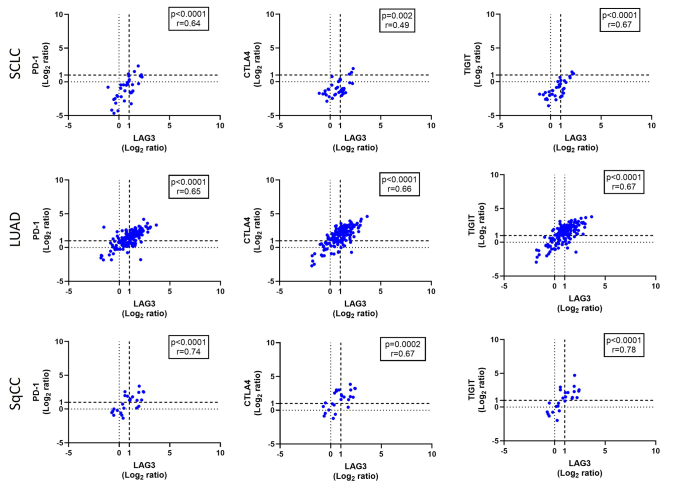


**Supplementary Data 3:**
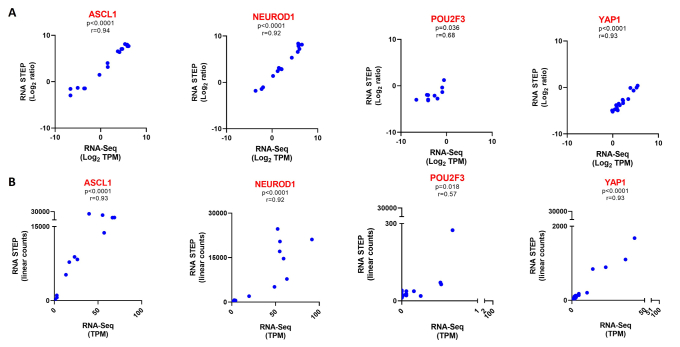


**Supplementary Data 6:**
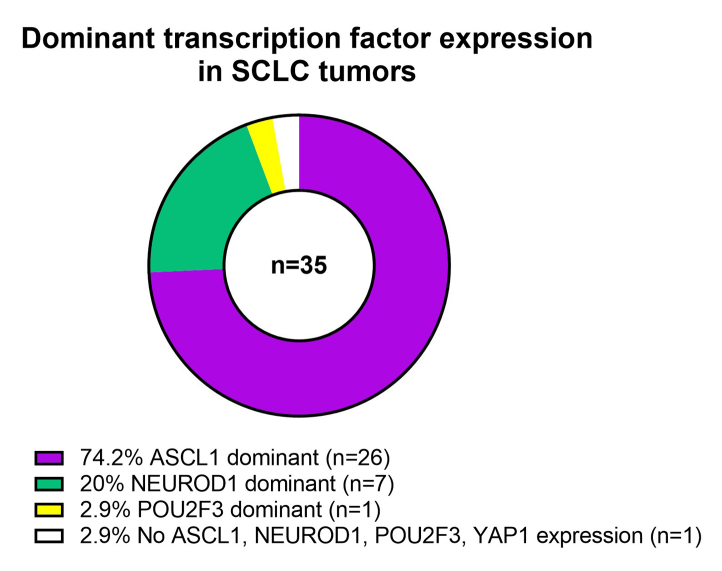


**Supplementary Data 7:**