# Supplementary material for: Unveiling the Molecular Features of SCLC With a Clinical RNA Expression Panel
Source: JTO Clin Res Rep. 2024 Aug 28;5(11):100723. doi: 10.1016/j.jtocrr.2024.100723 (PMC11459576; doi:10.1016/j.jtocrr.2024.100723)
Supplement: Supplementary Data 1 [file mmc1.docx]

**Supplemental Data 1. Supplementary methods.**

**RNA extraction**

Samples collected via the autopsy process with the RTD protocol were snap frozen within 15 minutes of collection and stored in liquid nitrogen until processing. Sections (4 um) from each frozen sample were stained with haematoxylin and eosin (H&E) and reviewed by a pathologist to evaluate adequacy. Thirty milligrams of tissue were macro dissected for tumor enrichment and used for total RNA extraction using the Qiagen RNeasy Tissue Kit (cat#69504, Germantown, MD) in accordance with manufacturer’s instructions. RNA quality was assessed using Qubit Fluorometric Quantification (Invitrogen, Waltham MA) and TapeStation 4200 (Agilent, Santa Clara, CA).

Formalin-fixed paraffin-embedded (FFPE) samples were used for clinical samples and clinical trial samples. Hematoxylin and eosin (H&E) stained slides were reviewed for adequacy and annotated for tumor enrichment by pathologists. mRNA extraction and purification were performed with the AllPrep® DNA/RNA FFPE protocol (Qiagen, Germantown, MD, Cat. No. 80234) using the automated QIAcube instrument (Qiagen, Germantown, MD). RNA concentration and quality were evaluated using the nanodrop ND-1000 spectrophotometer (Thermo Fisher Scientific, Waltham, MA). All samples yielded at least 40 ng of remnant mRNA for RNA STEP analysis of 204 genes (range 43-317 ng, tumor cell percentage 10-90%, see Fig. 1A for the assay targets).

**RNA Sequencing (RNA-Seq)**

RNA-Seq was performed using the Illumina TruSeq RNA Exome Library Preparation Kit (Illumina Inc., San Diego, CA) according to the manufacturer’s protocol.  Briefly, following evaluation of RNA quality with Agilent TapeStation  (Agilent Technologies, Santa Clara, CA) results and quantitation with the Qubit RNA BR Assay Kit (ThermoFisher Scientific, Waltham, MA), 100 ng of RNA was used as input into the initial divalent cation fragmentation.   cDNA libraries were generated according to the protocol and reviewed for quality and quantity using the Qubit dsDNA Assay Kit (ThermoFisher Scientific, Waltham, MA) and the BioAnalyzer DNA 1000 Chip (Agilent Technologies, Santa Clara, CA).  The libraries were normalized, pooled, and enriched twice using the provided Illumina exome probes.  Final library pools were reviewed for initial quantity and quality using the Qubit dsDNA Assay Kit and the BioAnalyzer High Sensitivity DNA Chip.  Following library quantitation with the Kapa Library Quantification Kits for NGS (Roche Sequencing, Pleasanton, CA), the libraries were sequenced on the NovaSeq 6000 (Illumina, Inc, San Diego, CA) to generate approximately 100M million pairs of 100-base reads per sample. A threshold of Transcripts per Million (TPM) value greater than 1 was employed to designate high gene expression in the RNA-Seq data, facilitating analyses of intra-tumoral and intra-patient heterogeneity.

**Immunohistochemistry (IHC)**

Representative whole sections of FFPE tissue were subjected to immunohistochemical (IHC) staining with antibodies for the following markers: ASCL1 (clone: E5S4Q, Cell Signaling), NEUROD1 (clone: EPR20766, Abcam), POU2F3 (polyclonal, Novus Biologicals), and YAP1 (clone: EP1674Y, Abcam). Nuclear staining was considered as positive and H scores were calculated by multiplying the percentage of positive cells (0-100) by the staining intensity (1-3).

**Statistical analyses**

As a first step, the RNA expression data was assessed to determine whether the data followed a normal distribution with the Shapiro-Wilk and the Kolmogorov-Smirnov normality tests. For normally distributed data, Spearman correlation analysis was performed to explore potential associations between sets of continuous or ordinal variables. Correlation coefficients and associated p-values were calculated to determine the statistical significance of these correlations. For non-normally distributed data the Mann-Whitney U test was applied to assess whether there were statistically significant differences between groups. The test provided insights into whether there were significant disparities between two groups for the variable of interest. P-values were calculated to determine the significance of these differences. All statistical analyses were performed using GraphPad Prism version 9.3.1 and IBM SPSS Statistics version 26. The Holm method was used to adjust p-values for multiple comparisons. A significance level (alpha) of <0.0001 was used to determine statistical significance. Graphical visualizations were performed with GraphPad Prism version 9.3.1.
