# Supplementary material for: Unveiling the Molecular Features of SCLC With a Clinical RNA Expression Panel
Source: JTO Clin Res Rep. 2024 Aug 28;5(11):100723. doi: 10.1016/j.jtocrr.2024.100723 (PMC11459576; doi:10.1016/j.jtocrr.2024.100723)
Supplement: Supplementary Material [file mmc8.docx]

Supplemental Data 1. Supplementary methods. RNA extraction, RNA-Seq, IHC, and statistical analyses.

Supplemental Data 2. Clinicopathological Features, RNA-Seq, and RNA STEP Results for ASCL1, NEUROD1, POU2F3, and YAP1 in 17 SCLC Samples Tested Using Both Methods.

Supplemental Data 3. Comparison of RNA STEP and RNA-Seq results showed a statistically significant correlation for 4 transcriptional factors of SCLC (n=17). (A) Numbers in the x and y-axis are shown as Log_2_ values of RNA-Seq TPM values and Log_2_ ratio of RNA STEP, respectively. (B) Numbers in the x and y-axis display RNA-Seq TPM values and RNA STEP mRNA linear counts, respectively.

Supplemental Data 4. H scores and RNA STEP Log_2_ Ratios for ASCL1, NEUROD1, POU2F3 and YAP1 in 12 SCLC samples tested with immunohistochemistry (IHC) and RNA STEP.

Supplemental Data 5. RNA STEP Log_2_ Ratios for 204 Genes in 220 Lung Cancer Samples.

Supplemental Data 6. Dominant transcription factor expression in 35 SCLC samples by RNA STEP.

Supplemental Data 7. Comparison of ASCL1, NEUROD1, and POU2F3, expression levels in primary and metastatic tumors of 8 patients by RNA-Seq. The distinct expression profiles observed in primary and metastatic tissues highlight heterogeneity for #3, #7, #25, and #32.

Supplemental Data 8. Clinicopathological Features and RNA-Seq TPM Values of ASCL1, NEUROD1, POU2F3 in 71 Small Cell Lung Cancer Samples.

Supplemental Data 9. Mean and Median RNA STEP Log_2_ Ratios of 204 genes in SCLC and LUAD Samples, Along with Comparative Statistical Analysis Results.

Supplemental Data 10. Genes regulating inflammatory response showed decreased expression in SCLC compared to LUAD. Differences were statistically significant (p<0.0001) except for LAG3. The black bars represent the mean value. The dotted Supplementary File Captionsline splits higher expression from lower expression.

Supplemental Data 11. Gene expression (log2 ratios) by RNA STEP for genes corresponding to LAG3, PD-1, PD-L1 and CTLA4 in 35 small cell lung cancer samples.

Supplemental Data 12. Correlation of expression results (log2 ratios) for genes corresponding to LAG3, PD-1, PD-L1 and CTLA4 in lung cancers stratified by small cell, adenocarcinoma, and squamous subtypes.
